# The Rising Phoenix-Progesterone as the Main Target of the Medical Therapy for Leiomyoma

**DOI:** 10.1155/2017/4705164

**Published:** 2017-09-13

**Authors:** H. H. Chill, M. Safrai, A. Reuveni Salzman, A. Shushan

**Affiliations:** Department of Obstetrics and Gynecology, Hadassah-Hebrew University Medical Center, Jerusalem, Israel

## Abstract

Leiomyomas, also known as uterine fibroids, are a common benign tumor in women of reproductive age. These lesions disrupt the function of the uterus causing menorrhagia and pelvic pressure as well as reproductive disorders. These women pose a true challenge for clinicians in the attempt of choosing the suitable treatment for each patient. Patient's age, interest in fertility preservation, and leiomyoma location and size are all factors to be taken into account when deciding upon the preferable therapeutic option. For the past few decades, surgical treatment was the only reliable long-term treatment available. A variety of surgical approaches have been developed over the years but these developments have come at the expense of other treatment options. The classical medical treatment includes gonadotropin-releasing hormone (GnRH) agonists and antagonists. These agents are well known for their limited clinical effect as well as their broad spectrum of side effects, inspiring a need for new pharmacological treatments. In recent years, promising results have been reported with the use of selective progesterone receptor modulators (SPRM). Long-term clinical trials have shown a reduction in bleeding and shrinkage of leiomyoma mass. These results instill hope for women suffering from symptomatic leiomyomas seeking an effective, long-term medical option for their condition.

## 1. Introduction

Uterine leiomyomas, also called fibroids, are the most common form of benign gynecological tumors [1, 2]. These are hormone sensitive tumors with a clonal origin, derived from myometrial smooth-muscle cells and connective tissue fibroblasts. Leiomyomas characteristically present as well encapsulated fibrotic tissue within the wall of the uterus occurring in 77% of all women with a higher incidence in African-American women [3–6].

**Figure 1:**
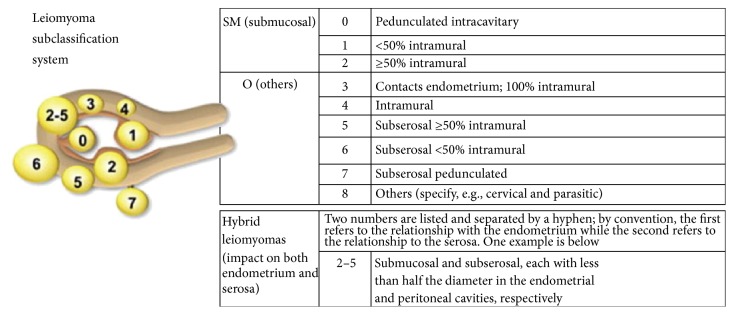
FIGO leiomyoma subclassification system.

Leiomyomas are commonly classified into 3 subgroups according to their location in the uterus: subserosal, intramural, and submucosal. A detailed classification system has been published by FIGO (International Federation of Gynecology and Obstetrics) (Figure 1), with specific attention to the fibroid's location [7].

The most recognized risk factors for the development of leiomyomas are early menarche, nulliparity, increased frequency of menses, history of dysmenorrhea, family history of leiomyomas, African descent, obesity, age (peak incidence at 40–50), and medical conditions such as diabetes and hypertension [8–11]. Behavioral attitudes such as diet with high consumption of meat or alcohol can also increase the risk, as opposed to smoking that decreases the risk [12–14].

In many cases leiomyomas are asymptomatic and are diagnosed incidentally on clinical examination or imaging. Only 20–50% of women suffer from a variety of symptoms, usually in accordance with the location and size of the mass [15, 16]. The symptoms are sometimes significant and can be divided into different categories: menorrhagia, space occupying manifestations, and reproductive disorders [17–21]. Women suffering from symptomatic leiomyomas have a significant lower health related quality of life and productivity: 43% will suffer an impact on sexual life, 28% will suffer an impact in performance at work, and 27% will be affected by the symptoms as a social matter in relationship and family [10, 22]. An improvement in quality of life has been shown following leiomyoma treatment, emphasizing the great need for a wide spectrum of therapeutic options.

Until recently, despite a great deal of research involving investment of substantial resources the goal of finding an effective medical treatment has eluded the scientific community. Nowadays, uterine leiomyomas remain the primary indication for hysterectomy in women of reproductive age in America [23].

Recently, a major change and hope have emerged. Selective progesterone receptor modulators (SPRM) have been offered as effective medical therapy for leiomyomas, with minimal side effects and promising long-term results. In this paper, we review these new pharmacological modalities and the opportunities they offer to a large population of women in need of alternative medical treatments.

## 2. Etiology

Despite years of research the pathogenesis of leiomyomas remains unclear. Clearly, enhancement of extracellular matrix (ECM) deposition plays an important role in the formation of uterine fibroids [24]. Norian et al. hypothesized that mechanical stress may set in motion a cascade of events leading to excessive ECM deposition which may bring about formation of uterine fibroids [25]. Several molecular pathways as well as genetic factors have been suggested as key elements in the development of uterine fibroids and have evoked much debate regarding possible treatments for inhibiting uterine fibroid growth. Tyrosine kinase inhibitors (TKI), cyclin-dependant kinase (CDK) inhibitors, aromatase inhibitors, and antiproliferative agents are only a partial list of biological mechanisms targeted by pharmaceutical solutions for the treatment for uterine fibroids [26–29]. Unfortunately, though in theory most of these treatments have biological merit to them, clinical results have been disappointing.

Over the years estrogen was considered to be the main culprit responsible for their growth. Recent studies have made it clear that progesterone too is an important player in leiomyoma growth. The clinical observations that have traditionally supported the estrogen hypothesis also support the hypothesis that progesterone is involved in the pathogenesis of leiomyomas. Similar to estrogen levels, progesterone levels are elevated during the reproductive years, decreased during menopause, and suppressed during GnRH agonist therapy [30]. One of the first reports to connect between progesterone and leiomyomas was in 1949 when Segaloff et al. observed increased cellularity in the histologic structure of leiomyomas in 6 patients treated with 20 mg progesterone daily during 30–128 days [31]. Later, Tiltman showed a significantly higher mitotic activity in leiomyomas of woman who were treated with medroxyprogesterone acetate compared to an untreated group [32]. Kawaguchi et al. in their study investigated the influence of the menstrual cycle on the mitosis rate of uterine fibroids [33]. They reported a significantly higher mitotic count in the secretory phase, suggesting that fibroid growth is affected by progesterone. In another study Lamminen et al. compared proliferative activity of uterine fibroids of different women, showing that, in postmenopausal women without hormone replacement therapy (HRT) or with estrogen only as HRT, low proliferative activity was demonstrated [34]. On the other hand, postmenopausal woman treated with estrogen and progesterone as HRT showed a proliferating index equal to that observed in premenopausal women. Brandon et al. demonstrated that compared to adjacent myometrium there is an increase in progesterone receptor messenger ribonucleic acid expression, as well as progesterone receptor protein level in leiomyoma tissue [35]. In the same study a significantly higher rate of the proliferation antigen Ki-67 was found in leiomyoma tissue, suggesting that amplified progesterone-mediated signaling is instrumental in the abnormal growth of these tumors.

In addition to the biochemical and histological evidence supporting the role for progesterone in the pathogenesis of leiomyomas, there is compelling clinical evidence supporting this hypothesis. In 1961 Mixson and Hammond reported that norethynodrel causes rapid but reversible enlargement of uterine leiomyomas [36]. Friedman et al. as well as Carr et al. demonstrated that medroxyprogesterone acetate inhibits the ability of GnRH agonist-induced hypoestrogenism to shrink uterine leiomyomas [37, 38]. In another prospective trial Friedman et al. suggested that high-dose norethindrone can reverse the effectiveness of GnRH agonist-induced leiomyoma shrinkage in a dose-dependent action [39].

In 2013, Bulun suggested a new theory, showing the influence of smooth-muscle stem cells and progesterone in the development of leiomyomas [40]. Based on these assumptions it seems as though genetic defects on the cellular level of the myometrial smooth-muscle are key in leiomyoma formation. Point mutations in mediator complex subunit 12 (MED12) as well as in high-mobility group AT hook 2 (HMGA2) have been linked with uterine fibroid development and may be the preliminary step leading to tumorigenesis [41, 42]. The genetic changes installed by this pivotal incident may later lead to the modification of signal pathway transduction involving beta-catenin and tumor growth factor-beta (TGF-beta). These proteins are thought to regulate cell proliferation ultimately leading to clonal expansion and uterine fibroid growth. These smooth-muscle cells remain sensitive to estrogen and progesterone and are triggered during receptor activation by the appropriate ligand.

The receptor for progesterone presents a potential target for pharmacological treatment of leiomyomas. When activated it acts as an important transcription factor for uterine fibroid growth [43]. When bound by the antiprogestin RU-486 the progesterone receptor begins a series of events ending in the increase of Kruppel-like factor 11 (KLF11). Increased levels of this tumor suppressor gene have been linked to inhibition of fibroid proliferation [44]. At the cellular nuclear level binding of progesterone to the progesterone receptor has also been shown to increase levels of the antiapoptotic protein B cell lymphoma-2 (BCL2) which in turn stunts cell death and leads to fibroid growth [45].

The full effect of the progesterone-progesterone receptor complex on stem cells as well as on differentiated cells in uterine fibroids is still poorly understood. It is suspected that binding of progesterone to the progesterone receptor brings on changes at the genetic and epigenetic level leading to propagation and proliferation of these benign tumors [40]. Due to the pivotal role of progesterone in the pathogenesis of leiomyoma growth, researchers as well as pharmaceutical companies have focused on finding compounds that might inhibit its effect. These efforts have brought forth the selective progesterone receptor modulators (SPRM) which so far have shown promising results.

## 3. Surgical Therapy

Choosing the appropriate treatment for uterine fibroids is not an easy task. Many parameters need to be taken into account including patient age, desire for fertility preservation, and the ability to undergo surgery [46–48]. To date, surgery remains the main treatment option for symptomatic women with uterine leiomyomas [49, 50]. Surgical treatment options include hysterectomy, myomectomy by laparoscopy, robotic surgery, or laparotomy as well as myomectomy by hysteroscopy. Prospective trials regarding surgical techniques and long-term outcomes with evaluation of symptoms are scarce making it difficult to recommend one treatment option over the other. The risks and benefits for each treatment option need to be presented to the patient enabling her to reach an informed decision with proper coordination of expectations.

Albeit the many surgical techniques available today, hysterectomy still remains the definitive treatment option for uterine fibroids. Suitable for patients for whom fertility is no longer an issue to be taken into account, hysterectomy offers low reintervention rates as well as high rates of symptom relief [51]. Hysterectomy does have some notable downsides. Published in 1994, the Main's Women's Health Study mentioned that only 72% of women reported improvement in symptoms caused by uterine fibroids [52]. In other studies abdominal hysterectomy was shown to correlate with higher rates of major complications compared to other invasive treatments such as uterine artery embolization [53, 54]. Hysterectomy no doubt will continue to be the treatment of choice for certain women though there is place for randomized controlled studies with long-term follow-up which will hopefully help assess the true value of this procedure.

In cases where fertility preservation is desired myomectomy remains the treatment of choice [55]. The abdominal approach for this procedure includes laparotomy and laparoscopy as well as robotic methods or when possible hysteroscopic myomectomy. Though considered technically challenging, laparoscopic myomectomy presents several advantages when compared to open myomectomy. Donnez et al. as well as several others showed faster recovery with less postoperative morbidity for patients undergoing laparoscopic myomectomy compared to the open approach. These advantages did not come at the expense of reproductive outcomes as well as recurrence rate which were similar for the two procedures [56, 57].

## 4. Medical Therapy

### 4.1. Current Medical Therapy

Over the years various medical treatments have been suggested based on the biological understanding of fibroid growth. Most treatments to this day have fallen short in giving a true long-term solution for women suffering from uterine fibroids. Two of the most common therapies are GnRH agonists or antagonists and aromatase inhibitors.

#### 4.1.1. GnRh Agonists and Antagonists

Until recently, GnRH agonists have been the most efficient pharmacological treatment for leiomyomas. GnRH agonists have a direct action on the pituitary, inducing downregulation and desensitization of the GnRH receptors, producing a hypogonadotropic state with consequent reduction in estradiol and progesterone [16]. GnRH agonists were found to decrease uterine bleeding, improve hematologic parameters, manage symptoms of menometrorrhagia, dysmenorrhea, and pelvic discomfort, and reduce uterine and leiomyoma size [58]. Nevertheless, this treatment cannot be given for a long period of time due to the many side effects that accompany it including bone loss, hot flashes, sleep disturbance, vaginal dryness, myalgia, arthralgia, and possible impairment of mood and cognition [37].

A review published in 2015 found low to moderate quality evidence that add-back therapy with tibolone, raloxifene, estriol, and ipriflavone helps to preserve bone density and that medroxyprogesterone acetate (MPA) and tibolone may reduce vasomotor symptoms. Larger uterine volume was an adverse effect associated with some add-back therapies (MPA, tibolone, and conjugated estrogen) [59]. Upon cessation of treatment there is a resumption of menses and pretreatment uterine volume [60]. Numerous side effects and temporary benefit have caused GnRH agonists to be mainly used in the preoperative setup. A systemic review found that the use of GnRH agonists for three to four months prior to fibroid surgery reduces both uterine volume and fibroid size. GnRh agonists are beneficial in the correction of preoperative iron deficiency anemia (if present) and reduce intraoperative blood loss. If uterine size is such that a midline incision is planned, this can be avoided in many women with the use of GnRH agonist. For women undergoing hysterectomy, a vaginal procedure is more likely following the use of these agents [60]. Another drawback of this therapy is that prior to the downregulation of the GnRH receptors there is an increase in estrogen level (flare-up) that might aggravate symptoms.

GnRH-antagonists achieve similar clinical results as the agonists but with more rapid onset due to the lack of initial flare-up observed with GnRH agonists. However, these agents are not available as long-term treatments, require daily injections, and have not been adopted as a common therapy for leiomyomas [61].

#### 4.1.2. Aromatase Inhibitors

The inhibition of the aromatase enzyme has been speculated to be a key mechanism in regulating hormone-dependent fibroid growth by inhibiting the production of estradiol. Estradiol, through the estrogen receptor *α*, induces the production of progesterone receptor which is essential for the response of fibroid tissue to progesterone; this response includes increased cell survival, cell proliferation, and enhancement of extracellular matrix [40]. Yet, a recent Cochrane review on the use of aromatase inhibitors concluded that there was no evidence to support the use of these agents as medical therapy for treating uterine fibroids [62].

### 4.2. Selective Progesterone Receptor Modulators (SPRM)

**Figure 2:**
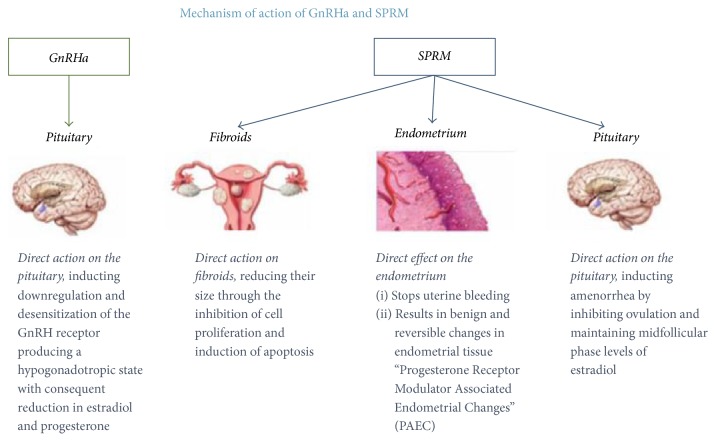
Mode of action of GnRH agonists and SPRM (selective progesterone receptor modulators). GnRH agonists have a direct impact on the pituitary. SPRM have a direct impact on fibroids, endometrium, and the pituitary [16].

SPRM are a family of substances which are known to incorporate both an agonist and an antagonist response on the receptor for progesterone (Figure 2) [16]. This response is mediated by many coreceptors and cofactors and has been shown to bare a favorable effect on the growth and development of leiomyomas [43, 63, 64]. This rationale has led pharmaceutical companies to invest in the research of these compounds leading to an array of products meant to stunt the growth of leiomyomas. In a recent publication we elaborate on their great potential and the important role these compounds may play in the near future [65]. Asoprisnil, mifepristone, and ulipristal acetate are a few examples of medications that were shown to be effective in decreasing the size of leiomyomas as well as reducing symptoms correlated with leiomyomas [66–70].

Ulipristal acetate is the most recent SPRM and has been under extensive investigation in the attempt to analyze its success in the treatment of uterine fibroids. This compound evokes an antiproliferative effect on leiomyoma cells as well as having a good safety profile with an easy to use regimen of one pill per day [71, 72]. Hence, it is easy to understand the enthusiasm in the scientific community regarding this potential treatment. In the PEARL I trial patients with symptomatic leiomyomas were treated with either placebo, 5 mg or 10 mg of ulipristal acetate for a duration of 13 weeks [73]. Results of this study showed a clear advantage for treatment with ulipristal acetate with control of menstrual bleeding in 92% of women who received a dose of 10 mg ulipristal acetate versus 19% in the placebo group. There was no difference between the groups regarding adverse effects. Leiomyoma volume, measured by magnetic resonance imaging, was reduced by a median reduction percentage of 21.2% for patients treated with 10 mg ulipristal acetate. The treatment's efficacy was shown with both objective (leiomyoma size) and subjective (patient discomfort) measures with encouraging results.

Later, a study was conducted comparing the efficacy of ulipristal acetate and a GnRH agonist. The PEARL II study, a randomized prospective trial, included women suffering from symptomatic uterine fibroids who received either an intramuscular injection of leuprolide acetate or treatment with ulipristal acetate (5 or 10 mg) [74]. Menstrual bleeding was controlled for patients who received 10 mg and 5 mg ulipristal acetate in 98 and 90 percent, respectively. Mean time to amenorrhea for these 2 groups was 5 and 7 days, respectively. For the leuprolide acetate group, control of menstrual bleeding was achieved in 89% with mean time to amenorrhea being 21 days. The difference in mean time to amenorrhea was statistically significant between the groups. Regarding reduction in uterine size, leuprolide acetate was superior when compared to ulipristal acetate. Hot flushes were a noteworthy side effect documented in 40% of patients treated with leuprolide acetate as opposed to 10% of women in the ulipristal acetate group. Conclusions of this study include ulipristal acetate being noninferior to leuprolide acetate with regard to the therapeutic effect on symptomatic leiomyomas with fewer side effects. In the following trial (PEARL III) ulipristal acetate was evaluated regarding its ability to induce a long-term effect for treatment of uterine fibroids. Two 12-week courses of treatment with ulipristal acetate 5 and 10 mg were administered to 451 patients enrolled in the study [64]. Amenorrhea was achieved in the 5 and 10 mg groups in 62 and 73 percent, respectively. During 2 treatment courses over 80% of patients achieved controlled bleeding. Median reductions from baseline in fibroid volume were 54 and 58 percent for the 5 mg and 10 mg groups, respectively. The treatment was well tolerated with under 5% of women abandoning treatment due to adverse effects. The investigators summarize that repeated 12-month treatment courses are effective in control of bleeding and reduction of fibroid size as well as improvement of quality of life (QOL) in patients suffering from symptomatic uterine fibroids [64, 71].

Fertility is a prominent issue in women with leiomyomas. Data regarding 21 patients who tried to get pregnant after UPA therapy (PEARL II and PEARL III trials) [75] showed that 15 women (71%) managed to conceive, resulting in a total of 18 pregnancies. Six women had a miscarriage and 12 pregnancies resulted in the live birth of 13 healthy babies. The high miscarriage rate may be explained by the median age of the population (38 years). Despite the hormonal changes expected during pregnancy no regrowth of leiomyomas was noted in pregnant women after cessation of UPA treatment.

Earlier this year, a new multicenter, prospective, noninterventional study (PREMYA) was published. A total of 1473 women with moderate to severe symptoms who received preoperative treatment with UPA (5 mg daily for 3 months) were enrolled. Data was collected every 3 months over a period of 12 months from the time treatment was discontinued. All patients were scheduled for surgery but only 38.8% finally underwent surgery. Physician assessment indicated that 60.1% of patients were either “much” or “very much” improved at 3 months.

A good safety profile was shown. Only one severe adverse effect was mentioned. It involved a diagnosis of leiomyosarcoma after hysterectomy. Only 56 (3.8%) patients stopped taking the medication due to side effects. This study reinforces previous results showing that quality of life and pain are highly improved by UPA treatment while maintaining a good safety profile.

In conclusion, SPRM are changing the way clinicians treat uterine fibroids. While surgical therapy remains the only definitive treatment, SPRM offer caregivers a viable option for treatment of this common pathology.

## References

[1] Donnez J., Jadoul P. (2002). What are the implications of myomas on fertility? A need for a debate?. *Human Reproduction*.

[2] Stewart E. A. (2001). Uterine fibroids. *The Lancet*.

[3] Marshall L. M., Spiegelman D., Barbieri R. L. (1997). Variation in the incidence of uterine leiomyoma among premenopausal women by age and race. *Obstetrics and Gynecology*.

[4] Baird D. D., Dunson D. B., Hill M. C., Cousins D., Schectman J. M. (2003). High cumulative incidence of uterine leiomyoma in black and white women: Ultrasound evidence. *American Journal of Obstetrics and Gynecology*.

[5] Chegini N. (2010). Proinflammatory and profibrotic mediators: Principal effectors of leiomyoma development as a fibrotic disorder. *Seminars in Reproductive Medicine*.

[6] Cramer S. F., Patel A. (1990). The frequency of uterine leiomyomas. *The American Journal of Clinical Pathology*.

[7] Munro M. G., Critchley H. O., Fraser I. S., FIGO Menstrual Disorders Working Group (2011). The FIGO classification of causes of abnormal uterine bleeding in the reproductive years. *Fertility and Sterility*.

[8] Faerstein E., Szklo M., Rosenshein N. B. (2001). Risk factors for uterine leiomyoma: A practice-based case-controls study. II. Atherogenic risk factors and potential sources of uterine irritation. *American Journal of Epidemiology*.

[9] Flake G. P., Andersen J., Dixon D. (2003). Etiology and pathogenesis of uterine leiomyomas: A review. *Environmental Health Perspectives*.

[10] Vilos G. A., Allaire C., Laberge P. Y. (2015). The management of uterine leiomyomas. *Journal of Obstetrics and Gynaecology Canada*.

[11] Sato F., Nishi M., Kudo R., Miyake H. (1998). Body fat distribution and uterine leiomyomas. *Journal of Epidemiology*.

[12] Wise L. A., Palmer J. R., Harlow B. L. (2004). Risk of uterine leiomyomata in relation to tobacco, alcohol and caffeine consumption in the Black Women's Health Study. *Human Reproduction*.

[13] Chiaffarino F., Parazzini F., La Vecchia C., Chatenoud L., Di Cintio E., Marsico S. (1999). Diet and uterine myomas. *Obstetrics and Gynecology*.

[14] Parazzini F., Negri E., La Vecchia C. (1996). Uterine myomas and smoking: Results from an Italian study. *Journal of Reproductive Medicine for the Obstetrician and Gynecologist*.

[15] Buttram V. C., Reiter R. C. (1981). Uterine leiomyomata: etiology, symptomatology, and management. *Fertility and Sterility*.

[16] Donnez J., Dolmans M.-M. (2016). Uterine fibroid management: From the present to the future. *Human Reproduction Update*.

[17] Parker W. H. (2007). Etiology, symptomatology, and diagnosis of uterine myomas. *Fertility and Sterility*.

[18] Pron G., Bennett J., Common A., Wall J., Asch M., Sniderman K. (2003). The Ontario Uterine Fibroid Embolization Trial. Part 2. Uterine fibroid reduction and symptom relief after uterine artery embolization for fibroids. *Fertility and Sterility*.

[19] Gupta S., Jose J., Manyonda I. (2008). Clinical presentation of fibroids. *Best Practice and Research: Clinical Obstetrics and Gynaecology*.

[20] Klatsky P. C., Tran N. D., Caughey A. B., Fujimoto V. Y. (2008). Fibroids and reproductive outcomes: a systematic literature review from conception to delivery. *American Journal of Obstetrics and Gynecology*.

[21] Pritts E. A., Parker W. H., Olive D. L. (2009). Fibroids and infertility: an updated systematic review of the evidence. *Fertility and Sterility*.

[22] Williams V. S. L., Jones G., Mauskopf J., Spalding J., Duchane J. (2006). Uterine fibroids: A review of health-related quality of life assessment. *Journal of Women's Health*.

[23] Fennessy F. M., Kong C. Y., Tempany C. M., Swan J. S. (2011). Quality-of-life assessment of fibroid treatment options and outcomes. *Radiology*.

[24] Luo X., Chegini N. (2008). The expression and potential regulatory function of MicroRNAs in the pathogenesis of leiomyoma. *Seminars in Reproductive Medicine*.

[25] Norian J. M., Owen C. M., Taboas J. (2012). Characterization of tissue biomechanics and mechanical signaling in uterine leiomyoma. *Matrix Biology*.

[26] Shushan A., Rojansky N., Laufer N. (2004). The AG1478 tyrosine kinase inhibitor is an effective suppressor of leiomyoma cell growth. *Human Reproduction*.

[27] Shime H., Kariya M., Orii A. (2002). Tranilast inhibits the proliferation of uterine leiomyoma cells in vitro through G1 arrest associated with the induction of p21waf1 and p53. *Journal of Clinical Endocrinology and Metabolism*.

[28] Shushan A., Ben-Bassat H., Mishani E., Laufer N., Klein B. Y., Rojansky N. (2007). Inhibition of leiomyoma cell proliferation in vitro by genistein and the protein tyrosine kinase inhibitor TKS050. *Fertility and Sterility*.

[29] Bulun S. E., Imir G., Utsunomiya H. (2005). Aromatase in endometriosis and uterine leiomyomata. *Journal of Steroid Biochemistry and Molecular Biology*.

[30] Rein M. S., Barbieri R. L., Friedman A. J. (1995). Progesterone: a critical role in the pathogenesis of uterine myomas. *American Journal of Obstetrics and Gynecology*.

[31] Segaloff A., Weed J. C., Sternberg W. H., Parson W. (1949). The progesterone therapy of human uterine leiomyomas. *The Journal of clinical endocrinology and metabolism*.

[32] Tiltman A. J. (1985). The effect of progestins on the mitotic activity of uterine fibromyomas. *International Journal of Gynecological Pathology*.

[33] Kawaguchi K., Fujii S., Konishi I., Nanbu Y., Nonogaki H., Mori T. (1989). Mitotic activity in uterine leiomyomas during the menstrual cycle. *American Journal of Obstetrics and Gynecology*.

[34] Lamminen S., Rantala I., Helin H., Rorarius M., Tuimala R. (1992). Proliferative activity of human uterine leiomyoma cells as measured by automatic image analysis. *Gynecologic and Obstetric Investigation*.

[35] Brandon D. D., Bethea C. L., Strawn E. Y. (1993). Progesterone receptor messenger ribonucleic acid and protein are overexpressed in human uterine leiomyomas. *American Journal of Obstetrics and Gynecology*.

[36] Mixson W. T., Hammond D. O. (1961). Response of fibromyomas to a progestin. *American journal of obstetrics and gynecology*.

[37] Friedman A. J., Barbieri R. L., Doubilet P. M., Fine C., Schiff I. (1988). A randomized, double-blind trial of a gonadotropin releasing-hormone agonist (leuprolide) with or without medroxyprogesterone acetate in the treatment of leiomyomata uteri. *Fertility and Sterility*.

[38] Carr B. R., Marshburn P. B., Weatherall P. T. (1993). An evaluation of the effect of gonadotropin-releasing hormone analogs and medroxyprogesterone acetate on uterine leiomyomata volume by magnetic resonance imaging: A prospective, randomized, double blind, placebo-controlled, crossover trial. *Journal of Clinical Endocrinology and Metabolism*.

[39] Friedman A. J., Daly M., Juneau-Norcross M. (1993). A prospective, randomized trial of gonadotropin-releasing hormone agonist plus estrogen-progestin or progestin “add-back” regimens for women with leiomyomata uteri. *Journal of Clinical Endocrinology and Metabolism*.

[40] Bulun S. E. (2013). Uterine fibroids. *The New England Journal of Medicine*.

[41] Mäkinen N., Mehine M., Tolvanen J. (2011). MED12, the mediator complex subunit 12 gene, is mutated at high frequency in uterine leiomyomas. *Science*.

[42] Markowski D. N., Helmke B. M., Belge G. (2011). HMGA2 and p14Arf: Major roles in cellular senescence of fibroids and therapeutic implications. *Anticancer Research*.

[43] Kim J. J., Sefton E. C. (2012). The role of progesterone signaling in the pathogenesis of uterine leiomyoma. *Molecular and Cellular Endocrinology*.

[44] Yin P., Lin Z., Reierstad S. (2010). Transcription factor KLF11 integrates progesterone receptor signaling and proliferation in uterine leiomyoma cells. *Cancer Research*.

[45] Yin P., Lin Z., Cheng Y.-H. (2007). Progesterone Receptor Regulates Bcl-2 Gene Expression through Direct Binding to Its Promoter Region in Uterine Leiomyoma Cells. *Journal of Clinical Endocrinology & Metabolism*.

[46] American College of Obstetricians and Gynecologists (2008). ACOG Practice Bulletin No. 96: Alternatives to Hysterectomy in the Management of Leiomyomas. *Obstetrics & Gynecology*.

[47] Stewart E. A. (2015). Uterine fibroids. *The New England Journal of Medicine*.

[48] Lumsden M. A., Hamoodi I., Gupta J., Hickey M. (2015). Fibroids: Diagnosis and management. *BMJ (Online)*.

[49] Donnez O., Jadoul P., Squifflet J., Donnez J. (2009). A series of 3190 laparoscopic hysterectomies for benign disease from 1990 to 2006: Evaluation of complications compared with vaginal and abdominal procedures. *BJOG: An International Journal of Obstetrics and Gynaecology*.

[50] The Practice Committee of the American Society for Reproductive Medicine in collaboration with The Society of Reproductive Surgeons (2008). Myomas and reproductive function. *Fertility and Sterility*.

[51] Spies J. B., Bradley L. D., Guido L. (2010). Outcomes from leiomyoma therapies: comparison with normal controls. *Obstetrics & Gynecology*.

[52] Carlson K. J., Miller B. A., Fowler F. J. (1994). The Maine Women's Health Study: II. Outcomes of nonsurgical management of leiomyomas, abnormal bleeding, and chronic pelvic pain. *Obstetrics and Gynecology*.

[53] Pinto I., Chimeno P., Romo A. (2003). Uterine fibroids: Uterine artery embolization versus abdominal hysterectomy for treatment - A prospective, randomized, and controlled clinical trial. *Radiology*.

[54] Dutton S., Hirst A., McPherson K., Nicholson T., Maresh M. (2007). A UK multicentre retrospective cohort study comparing hysterectomy and uterine artery embolisation for the treatment of symptomatic uterine fibroids (HOPEFUL study): Main results on medium-term safety and efficacy. *BJOG: An International Journal of Obstetrics and Gynaecology*.

[55] Segars J. H., Parrott E. C., Nagel J. D. (2014). Proceedings from the third national institutes of health international congress on advances in uterine leiomyoma research: Comprehensive review, conference summary and future recommendations. *Human Reproduction Update*.

[56] Donnez J., Donnez O., Dolmans M.-M. (2014). With the advent of selective progesterone receptor modulators, what is the place of myoma surgery in current practice?. *Fertility and Sterility*.

[57] Bhave Chittawar P., Franik S., Pouwer A. W., Farquhar C. (2014). Minimally invasive surgical techniques versus open myomectomy for uterine fibroids. *The Cochrane Database of Systematic Reviews*.

[58] Minaguchi H., Wong J. M., Snabes M. C. (2000). Clinical use of nafarelin in the treatment of leiomyomas: A review of the literature. *Journal of Reproductive Medicine for the Obstetrician and Gynecologist*.

[59] Moroni R. M., Martins W. P., Ferriani R. A. (2015). Add-back therapy with GnRH analogues for uterine fibroids. *The Cochrane database of systematic reviews*.

[60] Lethaby A., Vollenhoven B., Sowter M. (2002). Efficacy of pre-operative gonadotrophin hormone releasing analogues for women with uterine fibroids undergoing hysterectomy or myomectomy: a systematic review. *BJOG: An International Journal of Obstetrics and Gynaecology*.

[61] Flierman P. A., Oberyé J. J. L., Van Der Hulst V. P. M., De Blok S. (2005). Rapid reduction of leiomyoma volume during treatment with the GnRH antagonist ganirelix. *BJOG: An International Journal of Obstetrics and Gynaecology*.

[62] Song H., Lu D., Navaratnam K., Shi G. (2013). Aromatase inhibitors for uterine fibroids. *The Cochrane Database of Systematic Reviews*.

[63] Bouchard P., Chabbert-Buffet N., Fauser B. C. J. M. (2011). Selective progesterone receptor modulators in reproductive medicine: Pharmacology, clinical efficacy and safety. *Fertility and Sterility*.

[64] Donnez J., Vázquez F., Tomaszewski J. (2014). Long-term treatment of uterine fibroids with ulipristal acetate. *Fertility and Sterility*.

[65] Safrai M., Chill H. H., Reuveni Salzman A., Shushan A. (2017). Selective Progesterone Receptor Modulators for the Treatment of Uterine Leiomyomas. *Obstetrics & Gynecology*.

[66] Spitz I. M. (2009). Clinical utility of progesterone receptor modulators and their effect on the endometrium. *Current Opinion in Obstetrics and Gynecology*.

[67] Fiscella K., Eisinger S. H., Meldrum S., Feng C., Fisher S. G., Guzick D. S. (2006). Effect of mifepristone for symptomatic leiomyomata on quality of life and uterine size: a randomized controlled trial. *Obstetrics and Gynecology*.

[68] Wilkens J., Chwalisz K., Han C. (2008). Effects of the selective progesterone receptor modulator asoprisnil on uterine artery blood flow, ovarian activity, and clinical symptoms in patients with uterine leiomyomata scheduled for hysterectomy. *Journal of Clinical Endocrinology and Metabolism*.

[69] Nieman L. K., Blocker W., Nansel T. (2011). Efficacy and tolerability of CDB-2914 treatment for symptomatic uterine fibroids: A randomized, double-blind, placebo-controlled, phase IIb study. *Fertility and Sterility*.

[70] Shen Q., Hua Y., Jiang W., Zhang W., Chen M., Zhu X. (2013). Effects of mifepristone on uterine leiomyoma in premenopausal women: A meta-analysis. *Fertility and Sterility*.

[71] Horak P., Mara M., Dundr P. (2012). Effect of a selective progesterone receptor modulator on induction of apoptosis in uterine fibroids in vivo. *International Journal of Endocrinology*.

[72] Pohl O., Osterloh I., Gotteland J.-P. (2013). Ulipristal acetate - Safety and pharmacokinetics following multiple doses of 10-50 mg per day. *Journal of Clinical Pharmacy and Therapeutics*.

[73] Donnez J., Tatarchuk T. F., Bouchard P. (2012). Ulipristal acetate versus placebo for fibroid treatment before surgery. *The New England Journal of Medicine*.

[74] Donnez J., Tomaszewski J., Vázquez F. (2012). Ulipristal acetate versus leuprolide acetate for uterine fibroids. *The New England Journal of Medicine*.

[75] Luyckx M., Squifflet J.-L., Jadoul P., Votino R., Dolmans M.-M., Donnez J. (2014). First series of 18 pregnancies after ulipristal acetate treatment for uterine fibroids. *Fertility and Sterility*.

